# Performance of a rapid immuno-chromatographic test (Schistosoma ICT IgG-IgM) for detecting *Schistosoma*-specific antibodies in sera of endemic and non-endemic populations

**DOI:** 10.1371/journal.pntd.0010463

**Published:** 2022-05-27

**Authors:** Julie Hoermann, Esther Kuenzli, Carmen Schaefer, Daniel H. Paris, Silja Bühler, Peter Odermatt, Somphou Sayasone, Andreas Neumayr, Beatrice Nickel

**Affiliations:** 1 Swiss Tropical and Public Health Institute, Basel, Switzerland; 2 University of Basel, Basel, Switzerland; 3 Division of Hygiene and Infectious Diseases, Institute for Hygiene and Environment, Hamburg, Germany; 4 Department of International Program for Health in the Tropics, Lao Tropical and Public Health Institute, Vientiane, Lao People’s Democratic Republic; 5 Department of Public Health and Tropical Medicine, College of Public Health, Medical and Veterinary Sciences, James Cook University, Queensland, Australia; University of Passo Fundo: Universidade de Passo Fundo, BRAZIL

## Abstract

**Background:**

Schistosomiasis, an acute and chronic parasitic disease caused by human pathogenic *Schistosoma* species, is a neglected tropical disease affecting more than 220 million people worldwide.

For diagnosis of schistosomiasis, stool and urine microscopy for egg detection is still the recommended method, however sensitivity of these methods is limited. Therefore, other methods like molecular detection of DNA in stool, detection of circulating cathodic antigen in urine or circulating anodic antigen in urine and serum, as well as serological tests have gained more attention. This study examines the sensitivity and specificity of a rapid diagnostic test based on immunochromatography (Schistosoma ICT IgG-IgM, LD Bio, Lyon, France) for simultaneous detection of specific IgG and IgM antibodies in serum, against *Schistosoma* spp. in endemic and non-endemic populations.

**Methodology/Principal findings:**

Frozen banked serum samples from patients with confirmed schistosomiasis, patients with other helminth infections, patients with seropositive rheumatoid arthritis and healthy blood donors were used to assess the sensitivity and the specificity of the Schistosoma ICT IgG-IgM rapid diagnostic test.

The test showed a sensitivity of 100% in patients with parasitologically confirmed schistosomiasis, irrespective of the species (*S*. *mansoni*, *S*. *haematobium*, *S*. *japonicum*, *S*. *mekongi*). In healthy blood donors and patients with rheumatoid factor positive rheumatoid arthritis from Europe, specificity was 100%. However, in serum samples of patients with other tissue invasive helminth infections, the test showed some cross-reactivity, resulting in a specificity of 85%.

**Conclusion/Significance:**

With its high sensitivity, the Schistosoma ICT IgG-IgM rapid diagnostic test is a suitable screening test for detection of *Schistosoma* specific antibodies, including *S*. *mekongi*. However, in populations with a high prevalence of co-infection with other tissue invasive helminths, positive results should be confirmed with other diagnostic assays due to the test’s imperfect specificity.

## Author summary

Schistosomiasis is a neglected tropical disease, affecting more than 220 million people worldwide. Stool and urine microscopy are still the recommended diagnostic methods of choice, despite their limited sensitivity. Immunological diagnosis of schistosomiasis is gaining more attention due to higher sensitivity. This study investigated the sensitivity and the specificity of a rapid diagnostic test based on immunochromatography (Schistosoma ICT IgG-IgM, LD Bio, Lyon, France) designed to detect antibodies against *Schistosoma* specific antigens. We found a sensitivity of 100% in patients with proven schistosomiasis, regardless of species. In healthy blood donors, as well as in patients with seropositive rheumatoid arthritis, we found a specificity of 100%. In patients with other helminth infections, the Schistosoma ICT IgG-IgM showed some cross-reactivity, resulting in a specificity of 85%. In conclusion, the Schistosoma ICT IgG-IgM appears to be a suitable screening tool for *Schistosoma* specific antibodies. Confirmatory testing should be performed in patients at risk of co-infection with other tissue invasive parasites

## Introduction

Schistosomiasis is a neglected tropical parasitic disease caused by human-pathogenic *Schistosoma* species affecting more than 220 million people in 78 countries worldwide and causing approximately 1.9 million disability-adjusted life years (DALYs) [1, 2]. There are six human pathogenic *Schistosoma* species. Besides the three most prevalent species *Schistosoma mansoni*, *S*. *haematobium*, and *S*. *japonicum* three less prevalent species, namely *S*. *intercalatum*, *S*. *guineensis*, and *S*. *mekongi* occur in geographically more restricted areas [3].

The detection of *Schistosoma* eggs in stool or urine samples by microscopy is still considered the recommended method of choice for diagnosing schistosomiasis [4, 5]. While the specificity of stool microscopy is 98.8–100%, the sensitivity varies with the intensity of infection, the number of examined samples and the investigated amount of sample specimen [5, 6]. A study in an endemic area of Madagascar found a sensitivity of 19.3% for stool microscopy with the sedimentation method [7]. Another study investigating the accuracy of different screening tests in immigrants and refugees from African countries in Italy found a sensitivity of 45–48% for stool and urine microscopy [4]. Eggs can be detected earliest 4–6 weeks after infection, sometimes even later [5, 8].

Polymerase chain reaction (PCR) from urine or stool and detection of circulating microRNAs or cell-free circulating DNA in serum are more sensitive techniques showing a sensitivity and specificity of >90% and 98–100%, respectively [5]. In addition, PCR allows diagnosis already at an earlier stage of the infection, when eggs are not yet excreted by the parasite [8–11]. However, PCR is expensive and resource intense, and therefore not routinely available in endemic areas.

Other molecular methods targeting *Schistosoma* antigens, e.g. circulating cathodic antigen (CCA) or circulating anodic antigen (CAA) have been successfully applied for diagnosing schistosomiasis [12–17]. CCA and CAA are released by the adult worms and can be detected in blood, urine or even sputum at about 3 weeks after infection [5, 18]. A study investigating the CCA urine dipstick test for *S*. *man*soni (POC-CCA) found a sensitivity of 29% and a specificity of 93–95% [4, 5]. However the POC-CCA showed limited specificity in case of pregnancy, urinary tract infections or in young children [19–21].

The detection of CAA has been shown to be considerably more sensitive than that of CCA detection, but the assay procedure is complex and no commercial CAA assay is available to date [13].

Due to the higher sensitivity, immunological tests (e.g. enzyme-linked immunosorbent assay [ELISA], immunofluorescence antibody test [IFAT], indirect hemagglutination test [IHA], immunochromatographic test [ICT], Westernblot [WB]) are considered a supporting tool adjunct to direct detection methods. The disadvantage of serological tests is their inability to distinguish between active and past infection (“sero scars”) and potential cross-reactivity with antibodies induced by other tissue helminth infections. The latter may be especially problematic within endemic areas where co-infection with other parasites is frequent [5, 22].

In recent years, the availability of commercial rapid diagnostic tests (RDT) / point-of-care (POC) tests for schistosomiasis has increased, allowing immunodiagnostic testing in the field and in the absence of laboratory infrastructure.

The Schistosoma ICT IgG-IgM POC test (LDBIO Diagnostics, Lyon, France) simultaneously detects IgG and IgM antibodies against *S*. *mansoni* and *S*. *haematobium* antigen, based on lateral flow methodology. According to the manufacturer, the test has a sensitivity of 95.8% and a specificity of 92.4% [23]. In two recent studies in African migrants arriving in Europe, this Schistosoma ICT IgG-IgM POC test showed a sensitivity of 94% [4, 24].

With our study, we aimed on assessing the Schistosoma ICT IgG-IgM POC test’s sensitivity in patients with *S*. *mekongi* infection and the test’s specificity/cross-reactivity in patients with other tissue invasive helminth infections, as well as rheumatoid factor-positive rheumatoid arthritis. To our knowledge, this is the first study evaluating the test performance for detection of *S*. *mekongi* antibodies, and, besides the manufacturer’s own study [23], for potential cross-reactivity in the case of co-infections with other tissue invasive helminths or the presence of rheumatoid factor autoantibodies.

## Materials and methods

### Ethics statement

Ethical clearance for using the anonymized serum and stool samples was granted by the ethics committee of Northwest and Central Switzerland (Panel 1, 3 and 5: EKNZ UBE-15/22; Panel 4: EKNZ 257/13) and by the Lao National Ethics Committee for Health Research (Panel 2: 043/NECHR).

We evaluated the performance of the commercially available Schistosoma ICT IgG-IgM POC test (LDBIO diagnostics, Lyon, France) at the Diagnostic Center of the Swiss Tropical and Public Health Institute (Swiss TPH). The immunochromatographic test cassettes consist of nitrocellulose strips coated with purified antigen from crude lysate of *S*. *mansoni* adult worms for detection of specific antibodies. The test was performed according to the manufacturer’s instructions. In brief, test cassettes were brought to room temperature, 30 μl of serum was applied to the sample pad followed by 3 drops of kit-eluent. The results were read after 20 minutes by two independent observers blinded to any information linked to the samples. The test was judged positive if the control band and the test band were visible, negative if only the control band appeared.

We retrospectively tested five different serum panels from anonymized stored residual samples (S1 and S2 Figs):

‑ Panel 1: 39 sera from patients with microscopically confirmed (by egg detection in stool, urine or tissue biopsy) *Schistosoma mansoni* (n = 20), *S*. *haematobium* (n = 18) and *S*. *japonicum* (n = 1) infection diagnosed at the Swiss TPH. Stool analysis was performed according to published protocols with the sedimentation technique [12]. Based on the clinical symptoms and travel history, only stool microscopy or stool and urine microscopy were performed. However double infections can not completely be excluded.
‑ Panel 2: 35 sera from patients with microscopically confirmed *S*. *mekongi* infection (by detection of eggs in stool) collected during a Swiss TPH-Lao TPHI field study in Champasak district, Laos [25],
‑ Panel 3: 20 sera of healthy Swiss blood donors without known travel history to endemic areas,
‑ Panel 4: 20 sera from Swiss patients with rheumatoid factor-positive rheumatoid arthritis collected in context of an unrelated previous study [26] without known recent travel history to endemic areas, and
‑ Panel 5: 80 sera from patients who were tested negative for *Schistosoma*-specific antibodies, but positive for antibodies against other tissue invasive helminths at the Swiss TPH.

The sera of panel 5 consisted of the following samples:

- 70 samples which tested positive in one of the Swiss TPH in-house serological screening panel for tissue invasive parasites, consisting of six ELISAs (detecting antibodies against *Echinococcus* spp., *Fasciola hepatica*, *Filaria* spp., *Strongyloides stercoralis*, *Toxocara canis*, and *Trichinella spiralis*) and which also showed a positive result in the respective confirmatory assay (IHA: *Echinococcus* spp.; IFAT: *F*. *hepatica*, *Filaria* spp., *T*. *spiralis*; ELISA based on another antigen preparation: *Strongyloides papillosus*). In the case of an inconclusive or positive ELISA result for *Toxocara*, the identical ELISA was repeated since no alternative assay was available. The used positive reference sera for *Filaria* spp. and *Strongyloides* were from patients with parasitologically confirmed infections. Parasitological confirmation by microscopy, biopsy, or PCR was available for all *Strongyloides* and *Filaria* sera, and two *Fasciola* sera. For other infections and in the case of more than one positive test result in the serology panel, the most likely infection was defined based on the intensity of optical density (OD) values, the known typical cross-reactivity pattern among the assays, the clinical signs and symptoms, as well as the patient’s exposition history. The results of the screening ELISAs and the respective confirmatory assays of panel 5 are shown in S1 Table in the supplementary material.

- 5 serum samples each, which tested positive for *Taenia solium* cysticercosis or for *Angiostrongylus* spp. antibodies by Western Blots, confirmed by matching clinical symptoms and/or imaging results. The cysticercosis Western Blot is based on detection of antibodies to one or more of 7 purified glycoproteins (50, 39–42, 24, 18, 14, 13 kDa) according to Tsang et al., 1989 [27]. The test for *Angiostrongylus cantonensis* antibodies is based on purified proteins from adult worm lysate and detects specific reactivity at 31 kDa [28].

All serum samples except of panel 4 were tested by two *Schistosoma*-specific in-house ELISAs, one detecting antibodies against adult worm antigen extract (AWE) and one detecting antibodies against soluble egg antigen (SEA) [14]. Any positive or inconclusive result in one of the two screening ELISAs was confirmed or refuted by a consecutively performed in-house IFAT based on adult worm preparation [14]. A serum sample was considered negative if both screening ELISAs were negative. All serum samples of panel 1, 2, 3 and 5 were also tested on the serological screening panel for other tissue invasive parasites, consisting of six ELISAs (detecting antibodies against *Echinococcus* spp., *Fasciola hepatica*, *Filaria* spp., *Strongyloides stercoralis*, *Toxocara canis*, and *Trichinella spiralis*). In panel 1, three sera showed a positive Toxocara serology, in addition one serum a positive *Strongyloides* serology. In panel 2, 15 sera also showed a positive *Strongyloides* serology, 8 showed a positive *Filaria* serology and 6 showed a positive *Toxocara* serology. All other serologic tests were negative. As panel 2 sera were collected in an endemic region, co-infections with other helminths are very likely. As all *Schistosoma* sera of panel 1 and 2 were confirmed by egg detection or positive PCR it was assumed that *Schistosoma* specific antibodies are present in the sample.

All serum samples were stored at -80°C at the Swiss TPH and were thawed on the day of testing.

Sensitivity was calculated by dividing the number of ICT IgG-IgM POC positive tests by the corresponding number of the tested *Schistosoma* sera from Panel 1 and 2. Specificity was calculated by dividing the number of ICT IgG-IgM POC negative tests by the corresponding number of tested blood donor sera, rheumatoid factor positive samples and tissue invasive helminth samples, respectively.

## Results

**Fig 1:**
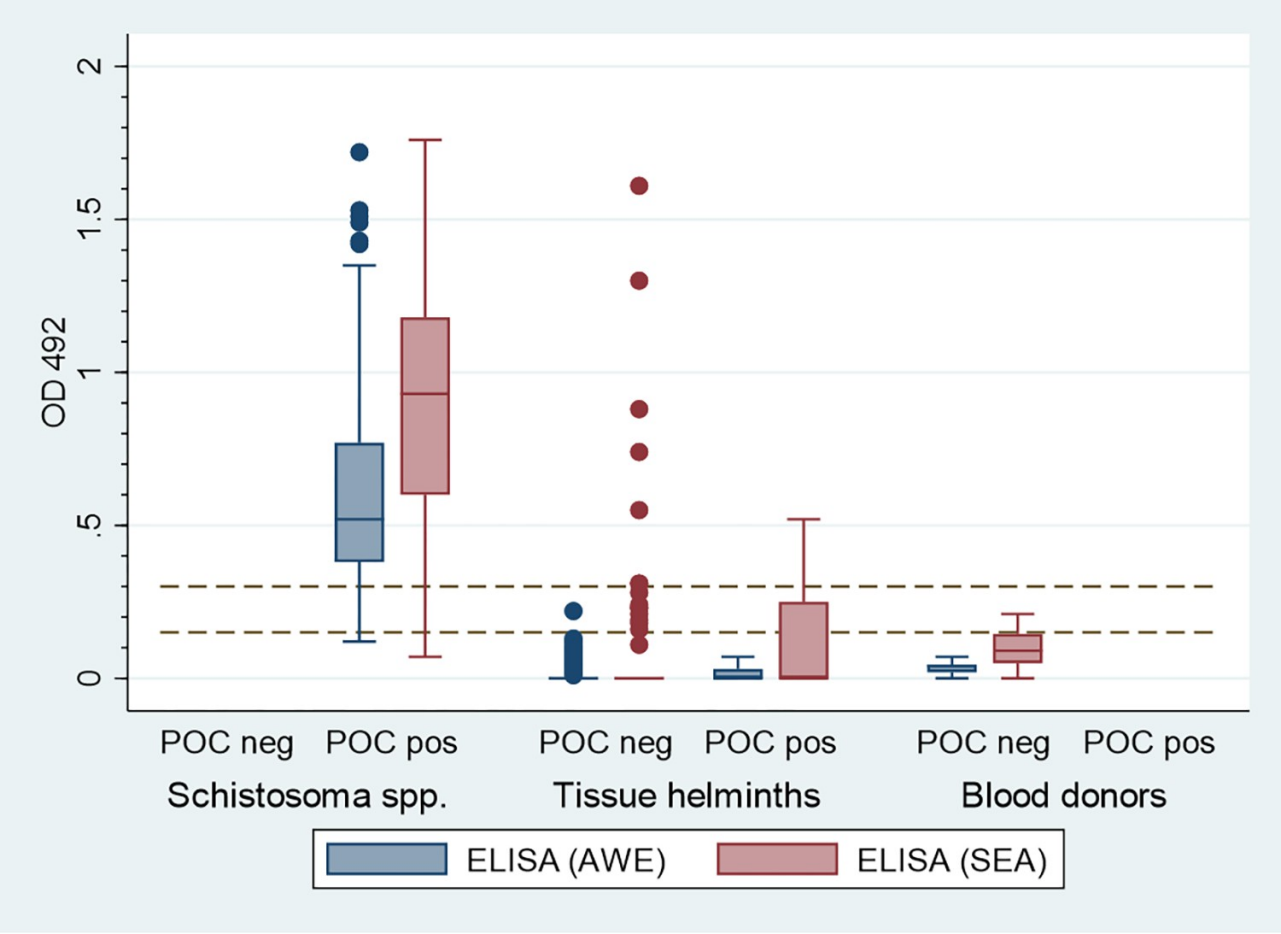
*Schistosoma* spp. (n = 74), tissue helminth (n = 80) and blood donor (n = 20) sera. OD 492 values of Adult antigen ELISA (AWE) and egg antigen ELISA (SEA) plotted for serum panels with ICT IgG-IgM POC negative and positive results. ELISA (AWE) OD < 0.15 negative, OD 0.15–0.29 inconclusive, OD ≥ 0.3 positive. ELISA (SEA) OD < 0.3 negative, OD 0.3–0.59 inconclusive, OD ≥ 0.6 positive. Dotted lines show the two cutoff values of the ELISAs.

The performance of the ICT IgG-IgM POC test with the five tested serum panels is shown in Table 1 and Fig 1. In total, 74 *Schistosoma* positive reference serum samples were tested with the ICT IgG-IgM POC test. All 74 samples showed a positive result for IgG/IgM, resulting in a sensitivity of 100% for all *Schistosoma* species.

**Table 1:** Performance of the ICT IgG-IgM POC test in the five tested serum panels.

Tested serum sample panel		ICT IgG-IgM POC test result		
	Number of samples	Positive N (%)	Negative N (%)	Sensitivity / Specificity (%)
** Panel 1:**				
*Schistosoma mansoni*	20	20 (100)	0 (0)	Sensitivity 100
*Schistosoma haematobium*	18	18 (100)	0 (0)	100
*Schistosoma japonicum*	1	1 (100)	0 (0)	
**Panel 2:**				
*Schistosoma mekongi*	35	35 (100)	0 (0)	Sensitivity 100
**Panel 3:**				
Blood donor samples	20	0 (0)	20 (100)	Specificity 100
**Panel 4:**				
Rheumatoid factor positive samples	20	0 (0)	20 (100)	Specificity 100
**Panel 5:**				
Tissue invasive helminth samples	80	12 (15)	68 (85)	Specificity 85
*Fasciola hepatica*	8	1 (13)	7 (88)	88
*Echinococcus* spp.	10	3 (30)	7 (70)	70
*Filaria* spp.	20	3 (15)	17 (85)	85
*Trichinella spiralis*	5	1 (20)	4 (80)	80
*Strongyloides stercoralis*	17	2 (12)	15 (88)	88
*Toxocara canis*	10	1 (10)	9 (90)	90
*Taenia solium* cysticercosis	5	1 (20)	4 (80)	80

A total of 40 serum samples of 20 healthy blood donors and of 20 patients with rheumatoid factor positive rheumatoid arthritis showed no reactive band on the ICT IgG-IgM POC test, resulting in a specificity of 100%.

Twelve of the 80 patient samples (15%) with other tissue invasive helminth infections showed a positive result in the ICT IgG-IgM POC test, resulting in an overall specificity of 85%. Cross-reactivity was mainly exhibited by *Echinococcus* spp., *Filaria* spp. and *Strongyloides* positive reference sera. Fig 1 shows the OD values (492 nm) of the two *Schistosoma*-specific in-house ELISAs, (AWE and SEA) for panel 1, 2, 3 and 5 sera plotted against ICT IgG-IgM POC results. Panel 1 and panel 2 also comprised *Schistosoma* sera with weak reactivity in the two in-house ELISAs which were all positive in the ICT IgG-IgM POC test. The S1 Table in the supplementary material shows the combined results of the screening ELISA, the confirmation tests, as well as the results of the ICT IgG-IgM POC test.

## Discussion

The recommended method for diagnosis of schistosomiasis is the detection of eggs in stool or urine specimens by microscopy, though eggs can also be detected in tissue samples. Nevertheless, the sensitivity is rather poor and always varies with intensity of infection and analyzed amount of sample [4–6]. The disadvantage of microscopy is a reduced sensitivity in settings with low endemicity and low infection intensity, respectively, whereas the strengths are low cost and operational advantages in resource-limited settings. Molecular methods targeting the parasite’s antigens or DNA of the parasite are more sensitive, however a sophisticated laboratory infrastructure is necessary [5, 12–17]. Immunodiagnostic methods with high sensitivity have been shown to be valuable tools for diagnosis of an infection but can fail in case of low infection intensity.

In our study, the ICT IgG-IgM POC test showed a sensitivity of 100% with 74 samples of confirmed *Schistosoma* spp. Infections, including 20 *S*. *mansoni*, 18 *S*. *haematobium*, 35 *S*. *mekongi* and 1 *S*. *japonicum* samples. The results show that the test is suitable for detection of *Schistosoma* infections of all tested species, not only *S*. *mansoni* and *S*. *haematobium*. The test simultaneously detects IgG and IgM antibodies leading to a higher sensitivity for also detecting recent infections, although we did not have information on the duration of illness matched to the samples. The good performance of the test for detection of *S*. *mekongi* antibodies is of special interest for endemic areas where control and elimination programs aim to combat transmission.

The results of our study confirm the good performance of the ICT IgG-IgM POC test for detecting antibodies against *S*. *haematobium* and *S*. *mansoni*, as indicated by the manufacturer. In addition, our results suggest that the test is suitable for diagnosing *S*. *mekongi* infections and possibly also *S*. *japonicum* infections. However, we had access to only one serum sample from a patient with proven *S*. *japonicum* infection.

To our knowledge, our study is the first one evaluating the ICT IgG-IgM POC test for its use to detect *S*. *mekongi* specific antibodies. This apparently good performance of the test may be of special interest for field studies in the Mekong area of Laos and Cambodia where *S*. *mekongi* is still endemic and control programs aim to eliminate transmission. Especially, as the RDT is easy to handle and does not require sophisticated laboratory infrastructure, which is often unavailable in rural areas of schistosomiasis endemicity.

It is known that rheumatoid factor can cause cross-reactivity in serological tests [29, 30]. However, this does not appear to be a major issue for the ICT IgG-IgM POC as no cross-reactivity was observed when sera of patients with rheumatoid factor-positive rheumatoid arthritis were tested. Likewise, the ICT IgG-IgM POC test did not show any cross-reactivity with serum samples of healthy blood donors.

Serological tests for tissue invasive helminths are known to show a certain degree of cross-reactivity with antibodies against other helminth antigens which negatively affects assay specificity [31–33]. According to the manufacturer of the ICT IgG-IgM POC test, possible cross-reactivity with *T*. *solium* cysticercosis and *Echinococcus* spp. sera has been described [23]. In our study, the ICT IgG-IgM POC test showed cross-reactivity with 15% of the tested sera from patients with other tissue invasive helminth infections, namely *Filaria* spp. (3/20), *Strongyloides stercoralis* (2/17), *Echinococcus* spp. (3/10), *Toxocara* (1/10), *T*. *solium* cysticercosis (1/5) and *Trichinella spiralis* (1/5) which suggests that the assay’s cross-reactivity is not limited to the parasite-specific antibodies indicated by the manufacturer, but may also extend to antibodies against other parasite species. In this regard, it has to be kept in mind that in most patients from endemic areas it is frequently difficult or even impossible to reliably exclude the presence of co-infections with other tissue invasive parasites. Especially, since low grade infections with tissue invasive parasites may not be provable by direct detection methods. Considering this obstacle and since we had no access to serum samples from Laotian individuals living in Laos outside *S*. *mekongi* endemic areas, we chose samples obtained from blood donations in Switzerland as most likely truly negative reference sera to assess the ICT IgG-IgM POC test’s specificity. Since formally the calculation of the ICT IgG-IgM POC test’s specificity for *S*. *mekongi* infections would have demanded using reference sera from healthy Laotians, we acknowledge that our specificity calculation based on healthy European blood donors as reference population is suboptimal.

In the same way it is difficult to obtain reliably true negative reference samples for the validation of serological assays, this is also problematic with regards to reliably true positive referrefence samples. The used reference sera of panel 5 were carefully selected based on corresponding positive serology in combination with matching clinical symptoms (e.g. *Trichinella*, *Toxocara*), parasitological confirmation (i.e. proof of infection by microscopic detection of microfilaria or *Strongyloides* larva in clinical samples) or matching imaging results in the case of *Echinococcus* spp., *T*. *solium* cysticercosis and *Fasciola* to ensure their validity. Nevertheless, for some reference sera a parasitological proof is missing. In addition, some positive reference samples exhibit reactivity in several immunoassays. Whether this observed reactivity represents cross-reactivity or may be due to coinfection can also not be proven beyond doubt. Although these limitations should be kept in mind, we consider it valid to conclude that the ICT IgG-IgM POC test apparently shows some cross-reactivity with other helminth antibodies and that the test’s overall specificity may be reduced in populations in which multiple helminth infections are common. Since our access to well characterized serum samples from patients with proven tissue invasive parasite infections was limited, a more extensive evaluation of the test by other laboratories would provide more insight.

Besides the above mentioned limitations the main limitation of our study is the overall low number of tested samples. The infection intensity of *Schistosoma* positive individuals could not be determined as no egg count was determined with the applied sedimentation method. Only few microscopy positive but Schistosoma-ELISA negative serum samples could be tested with the ICT IgG-IgM POC test. In addition we could not evaluate the performance of the ICT IgG-IgM POC test in low level infections which are only positive in PCR but negative in the ELISA.

Considering the very promising data, further evaluation of the ICT IgG-IgM POC test’s performance in larger studies would be desirable.

## Conclusion

The results of this study show that the ICT IgG-IgM POC test is a suitable tool for detection of antibodies directed against all *Schistosoma* species. The determined specificity of the ICT IgG-IgM POC was 100% with sera from healthy blood donors and from patients with rheumatoid factor positive rheumatoid arthritis. However, cross-reactivity was observed with serum samples from patients with various other tissue invasive helminth infections, leading to an overall specificity of 85% in this subgroup of patients.

Considering the test’s high specificity in populations lacking co-infections with other tissue invasive helminths, the ICT IgG-IgM POC test appears to be an ideal screening test for returning travelers. Although the ICT IgG-IgM POC test does not differentiate between past and current infections and cross-reactivity with other tissue invasive parasite specific antibodies may occur, its easy handling may still make the test attractive and valuable for epidemiological studies in schistosomiasis endemic areas.

## Supporting information

S1 STARD Checklist STARD checklist. Source: https://www.equator-network.org/reporting-guidelines/stard/ (download: 20 January 2022).
(PDF)

S1 Table Specificity assessment of the ICT IgG-IgM POC test (test panel 5). (PDF)

S1 Fig Diagram with *Schistosoma* positive Sera of Panel 1 and 2. (PDF)

S2 Fig Diagram with *Schistosoma* negative Sera of Panel 3, 4 and 5. (PDF)
