# Supplementary material for: Performance of a rapid immuno-chromatographic test (Schistosoma ICT IgG-IgM) for detecting Schistosoma-specific antibodies in sera of endemic and non-endemic populations
Source: PLoS Negl Trop Dis. 2022 May 27;16(5):e0010463. doi: 10.1371/journal.pntd.0010463 (PMC9212132; doi:10.1371/journal.pntd.0010463)
Supplement: S1 STARD Checklist — Source: https://www.equator-network.org/reporting-guidelines/stard/ (download: 20 January 2022). (PDF) [file pntd.0010463.s001.pdf]

Section & Topic	No	Item	Reported on page #
**TITLE OR ABSTRACT**			
	**1**	Identification as a study of diagnostic accuracy using at least one measure of accuracy (such as sensitivity, specificity, predictive values, or AUC)	Page 1, Title
**ABSTRACT**			
	**2**	Structured summary of study design, methods, results, and conclusions (for specific guidance, see STARD for Abstracts)	Page 2, Abstract
**INTRODUCTION**			
	**3**	Scientific and clinical background, including the intended use and clinical role of the index test	Introduction
	**4**	Study objectives and hypotheses	Introduction
**METHODS**			
Study design	**5**	Whether data collection was planned before the index test and reference standard were performed (prospective study) or after (retrospective study)	Material and Methods
Participants	**6**	Eligibility criteria	Material and Methods
	**7**	On what basis potentially eligible participants were identified (such as symptoms, results from previous tests, inclusion in registry)	Material and Methods
	**8**	Where and when potentially eligible participants were identified (setting, location and dates)	Not applicable, retrospective study
	**9**	Whether participants formed a consecutive, random or convenience series	Not applicable, samples from biobank
Test methods	**10a**	Index test, in sufficient detail to allow replication	Material and Methods
	**10b**	Reference standard, in sufficient detail to allow replication	Material and Methods
	**11**	Rationale for choosing the reference standard (if alternatives exist)	Introduction
	**12a**	Definition of and rationale for test positivity cut-offs or result categories of the index test, distinguishing pre-specified from exploratory	Material and Methods
	**12b**	Definition of and rationale for test positivity cut-offs or result categories of the reference standard, distinguishing pre-specified from exploratory	Microscopy egg detection
	**13a**	Whether clinical information and reference standard results were available to the performers/readers of the index test	Material and Methods
	**13b**	Whether clinical information and index test results were available to the assessors of the reference standard	Not applicable, retrospective study
Analysis	**14**	Methods for estimating or comparing measures of diagnostic accuracy	Material and Methods
	**15**	How indeterminate index test or reference standard results were handled	Not applicable, retrospective study
	**16**	How missing data on the index test and reference standard were handled	Material and Methods
	**17**	Any analyses of variability in diagnostic accuracy, distinguishing pre-specified from exploratory	Not applicable
	**18**	Intended sample size and how it was determined	Not applicable, retrospective study
**RESULTS**			
Participants	**19**	Flow of participants, using a diagram	Supplementary S1 Fig and S2 Fig
	**20**	Baseline demographic and clinical characteristics of participants	Material and Methods
	**21a**	Distribution of severity of disease in those with the target condition	Not applicable
	**21b**	Distribution of alternative diagnoses in those without the target condition	Not applicable
	**22**	Time interval and any clinical interventions between index test and reference standard	Not applicable
Test results	**23**	Cross tabulation of the index test results (or their distribution) by the results of the reference standard	Fig 1
	**24**	Estimates of diagnostic accuracy and their precision (such as 95% confidence intervals)	Results
	**25**	Any adverse events from performing the index test or the reference standard	Not applicable

cc BY SA

**DISCUSSION**			
	**26**	Study limitations, including sources of potential bias, statistical uncertainty, and generalisability	Discussion
	**27**	Implications for practice, including the intended use and clinical role of the index test	Discussion
**OTHER INFORMATION**			
	**28**	Registration number and name of registry	Not applicable
	**29**	Where the full study protocol can be accessed	Page 1, corr. author
	**30**	Sources of funding and other support; role of funders	No funding obtained

cc BY SA
