# Supplementary material for: Performance of a rapid immuno-chromatographic test (Schistosoma ICT IgG-IgM) for detecting Schistosoma-specific antibodies in sera of endemic and non-endemic populations
Source: PLoS Negl Trop Dis. 2022 May 27;16(5):e0010463. doi: 10.1371/journal.pntd.0010463 (PMC9212132; doi:10.1371/journal.pntd.0010463)
Supplement: S1 Table — (PDF) [file pntd.0010463.s002.pdf]

**S1 Table: Specificity assessment of the ICT IgG-IgM POC test (test panel 5).**

Testing of sera with the ICT IgG-IgM POC test, which are considered truly negative for *Schistosoma* specific antibodies (as determined by an in-house two-tier testing strategy) but considered truly positive for antibodies of various other tissue invasive parasites.

| | *Schistosoma* specific serologies | | | Serology for other tissue invasive parasite species | | | | | | | | | | | | | |
|---|---|---|---|---|---|---|---|---|---|---|---|---|---|---|---|---|---|
| **No. of tested serum sample** | ***Schistosoma* adult antigen ELISA** | ***Schistosoma* egg antigen ELISA** | ***Schistosoma* adult worm IFAT** | ***Trichinella* ELISA** | ***Trichinella* IHA** | ***Toxocara* ELISA [A]** | ***Echinococcus* ELISA** | ***Echinococcus* IHA** | ***Fasciola* ELISA** | ***Fasciola* IFAT** | ***Filaria* ELISA** | ***Filaria* IFAT** | ***Strongyloides* ELISA-1** | ***Strongyloides* ELISA-2 [A,B]** | ***Taenia solium* cysticercosis WB** | ***Angiostrongylus cantonensis* WB** | **ICT IgG-IgM POC test** |
| **1** | - | - | n.d. | - | n.d. | + | + | - | - | n.d. | + | +/- | +* | [A] | n.d. | n.d. | + |
| **2** | - | - | n.d. | - | n.d. | - | - | n.d. | - | n.d. | - | n.d. | +* | [A] | n.d. | n.d. | - |
| **3** | - | - | n.d. | - | n.d. | - | - | n.d. | - | n.d. | - | n.d. | +* | [A] | n.d. | n.d. | - |
| **4** | - | - | n.d. | - | n.d. | - | - | n.d. | - | n.d. | +/- | - | +/-* | [A] | n.d. | n.d. | - |
| **5** | - | - | n.d. | - | n.d. | - | - | n.d. | - | n.d. | - | n.d. | +/-* | [A] | n.d. | n.d. | - |
| **6** | - | - | n.d. | - | n.d. | - | - | n.d. | - | n.d. | - | n.d. | +* | [A] | n.d. | n.d. | - |
| **7** | - | - | n.d. | - | n.d. | - | + | - | - | n.d. | + | n.d. | +* | [A] | n.d. | n.d. | - |
| **8** | - | - | n.d. | - | n.d. | - | - | n.d. | - | n.d. | - | n.d. | +* | [A] | n.d. | n.d. | - |
| **9** | - | - | n.d. | - | n.d. | - | + | - | - | n.d. | + | n.d. | -* | [A] | n.d. | n.d. | - |
| **10** | - | +/- | - | - | n.d. | - | - | n.d. | - | n.d. | - | n.d. | +* | [A] | n.d. | n.d. | - |
| **11** | - | - | n.d. | - | n.d. | - | + | - | +/- | n.d. | + | n.d. | +* | [A] | + | n.d. | - |
| **12** | - | - | n.d. | - | n.d. | - | + | - | +/- | - | + | - | +* | [A] | n.d. | n.d. | - |
| **13** | - | - | n.d. | - | n.d. | - | - | n.d. | - | n.d. | - | n.d. | +* | [A] | n.d. | n.d. | + |
| **14** | - | - | n.d. | - | n.d. | - | - | n.d. | - | n.d. | - | n.d. | -* | [A] | n.d. | n.d. | - |
| **15** | - | - | n.d. | - | n.d. | - | - | n.d. | - | n.d. | - | n.d. | +/-* | +[B] | n.d. | n.d. | - |
| **16** | - | - | n.d. | - | n.d. | - | - | n.d. | - | n.d. | - | n.d. | +* | +[B] | n.d. | n.d. | - |
| **17** | - | +/- | - | - | n.d. | - | - | n.d. | - | n.d. | - | n.d. | +* | +[B] | n.d. | n.d. | - |
| **18** | - | - | n.d. | - | n.d. | - | + | - | +/-* | + | +/- | - | - | n.d. | n.d. | n.d. | - |
| **19** | - | - | n.d. | - | n.d. | - | - | n.d. | +* | + | - | n.d. | - | n.d. | n.d. | n.d. | - |
| **20** | - | - | n.d. | - | n.d. | - | - | n.d. | + | + | - | n.d. | - | n.d. | n.d. | n.d. | - |
| **21** | - | - | n.d. | - | n.d. | - | + | - | + | + | +/- | n.d. | - | n.d. | n.d. | n.d. | - |
| **22** | - | - | n.d. | - | n.d. | +/- | - | n.d. | + | + | - | n.d. | + | [A] | n.d. | n.d. | + |
| **23** | - | - | n.d. | + | n.d. | +/- | + | n.d. | + | + | + | n.d. | + | [A] | n.d. | n.d. | - |
| **24** | - | - | n.d. | - | n.d. | - | - | n.d. | + | + | + | n.d. | +/- | [A] | n.d. | n.d. | - |
| **25** | - | - | n.d. | - | n.d. | - | - | n.d. | + | + | - | n.d. | - | n.d. | n.d. | n.d. | - |
| **26** | - | - | n.d. | - | n.d. | - | - | n.d. | - | n.d. | +* | +/- | - | n.d. | n.d. | n.d. | - |
| **27** | - | - | n.d. | - | n.d. | - | + | - | - | n.d. | +* | + | - | n.d. | n.d. | n.d. | - |
| **28** | - | - | n.d. | - | n.d. | - | + | - | - | n.d. | +* | + | +/- | [A] | n.d. | n.d. | + |
| **29** | - | - | n.d. | - | n.d. | - | + | - | - | n.d. | +* | - | + | [A] | n.d. | n.d. | - |
| **30** | - | - | n.d. | - | n.d. | - | - | n.d. | - | n.d. | +* | - | - | n.d. | n.d. | n.d. | - |
| **31** | - | - | n.d. | - | n.d. | - | - | n.d. | - | n.d. | -* | - | + | [A] | n.d. | n.d. | + |
| **32** | - | - | n.d. | - | n.d. | - | - | n.d. | - | n.d. | +/-* | - | + | [A] | n.d. | n.d. | - |
| **33** | - | - | n.d. | - | n.d. | - | + | - | - | n.d. | +* | - | + | [A] | n.d. | n.d. | - |
| **34** | - | - | n.d. | - | n.d. | - | - | n.d. | - | n.d. | +* | +/- | - | n.d. | n.d. | n.d. | - |
| **35** | - | - | n.d. | - | n.d. | +/- | + | - | - | n.d. | +* | - | - | n.d. | n.d. | n.d. | - |
| **36** | - | - | n.d. | - | n.d. | - | + | - | - | n.d. | +* | +/- | - | n.d. | n.d. | n.d. | - |

| | | | | | | | | | | | | | | | | | |
|---|---|---|---|---|---|---|---|---|---|---|---|---|---|---|---|---|---|
| 37 | - | - | n.d. | - | n.d. | - | + | - | - | n.d. | +* | - | + | n.d. | n.d. | n.d. | - |
| 38 | - | - | n.d. | - | n.d. | +/- | + | - | - | n.d. | +* | +/- | - | n.d. | n.d. | n.d. | - |
| 39 | - | - | n.d. | - | n.d. | - | - | n.d. | - | n.d. | -* | n.d. | + | n.d. | n.d. | n.d. | - |
| 40 | - | - | n.d. | - | n.d. | - | + | - | - | n.d. | +* | - | + | n.d. | n.d. | n.d. | - |
| 41 | - | - | n.d. | - | n.d. | - | - | n.d. | - | n.d. | +* | +/- | - | n.d. | n.d. | n.d. | - |
| 42 | - | - | n.d. | - | n.d. | - | + | - | - | n.d. | +* | + | - | n.d. | n.d. | n.d. | - |
| 43 | - | - | n.d. | - | n.d. | - | + | - | - | n.d. | +* | + | +/- | [A] | n.d. | n.d. | + |
| 44 | - | - | n.d. | - | n.d. | - | - | n.d. | - | n.d. | +* | +/- | - | n.d. | n.d. | n.d. | - |
| 45 | - | - | n.d. | - | n.d. | - | + | - | - | n.d. | +* | + | +/- | - [B] | n.d. | n.d. | - |
| 46 | - | - | n.d. | - | n.d. | + | - | n.d. | - | n.d. | - | n.d. | - | n.d. | n.d. | n.d. | - |
| 47 | - | - | n.d. | - | n.d. | + | - | n.d. | - | n.d. | - | n.d. | - | n.d. | n.d. | n.d. | - |
| 48 | - | - | n.d. | - | n.d. | + | - | n.d. | - | n.d. | - | n.d. | - | n.d. | n.d. | n.d. | - |
| 49 | - | - | n.d. | - | n.d. | + | - | n.d. | - | n.d. | - | n.d. | - | n.d. | n.d. | n.d. | - |
| 50 | - | - | n.d. | - | n.d. | + | - | n.d. | - | n.d. | - | n.d. | - | n.d. | n.d. | n.d. | + |
| 51 | - | - | n.d. | - | n.d. | + | - | n.d. | - | n.d. | - | n.d. | - | n.d. | n.d. | n.d. | - |
| 52 | - | - | n.d. | - | n.d. | + | - | n.d. | - | n.d. | - | n.d. | - | n.d. | n.d. | n.d. | - |
| 53 | - | - | n.d. | - | n.d. | + | - | n.d. | - | n.d. | - | n.d. | - | n.d. | n.d. | n.d. | - |
| 54 | - | - | n.d. | - | n.d. | + | - | n.d. | - | n.d. | - | n.d. | - | n.d. | n.d. | n.d. | - |
| 55 | - | - | n.d. | - | n.d. | + | - | n.d. | - | n.d. | - | n.d. | - | n.d. | n.d. | n.d. | - |
| 56 | - | +/- | - | + | +/- | +/- | - | n.d. | - | n.d. | - | n.d. | - | n.d. | n.d. | n.d. | + |
| 57 | - | - | n.d. | +/- | + | - | - | n.d. | - | n.d. | - | n.d. | - | n.d. | n.d. | n.d. | - |
| 58 | - | - | n.d. | + | +/- | - | - | n.d. | - | n.d. | - | n.d. | - | n.d. | n.d. | n.d. | - |
| 59 | - | - | n.d. | + | + | + | - | n.d. | - | n.d. | - | n.d. | - | n.d. | n.d. | n.d. | - |
| 60 | - | - | n.d. | + | +/- | - | +/- | - | - | n.d. | + | +/- | - | n.d. | n.d. | n.d. | - |
| 61 | - | - | n.d. | - | n.d. | +/- | + | + | - | n.d. | + | +/- | - | n.d. | n.d. | n.d. | - |
| 62 | - | - | n.d. | - | n.d. | - | + | + | - | n.d. | +/- | n.d. | + | [A] | n.d. | n.d. | - |
| 63 | - | - | n.d. | - | n.d. | - | + | + | - | n.d. | + | n.d. | +/- | [A] | n.d. | n.d. | + |
| 64 | - | - | n.d. | - | n.d. | - | + | + | - | n.d. | - | n.d. | - | n.d. | n.d. | n.d. | - |
| 65 | - | - | n.d. | - | n.d. | - | + | + | - | n.d. | + | n.d. | +/- | [A] | n.d. | n.d. | + |
| 66 | - | - | n.d. | - | n.d. | - | + | + | - | n.d. | + | n.d. | +/- | [A] | n.d. | n.d. | - |
| 67 | - | - | n.d. | - | n.d. | - | + | + | - | n.d. | - | n.d. | - | n.d. | n.d. | n.d. | - |
| 68 | - | - | n.d. | - | n.d. | +/- | + | + | - | n.d. | + | n.d. | - | n.d. | n.d. | n.d. | - |
| 69 | - | - | n.d. | - | n.d. | - | + | + | - | n.d. | + | - | + | [A] | n.d. | n.d. | - |
| 70 | - | - | n.d. | - | n.d. | - | + | + | - | n.d. | +/- | n.d. | +/- | [A] | n.d. | n.d. | + |
| 71[a] | - | - | n.d. | - | n.d. | - | - | n.d. | - | n.d. | - | n.d. | - | n.d. | + | n.d. | - |
| 72[a] | - | - | n.d. | - | n.d. | - | - | n.d. | - | n.d. | - | n.d. | - | n.d. | + | n.d. | - |
| 73[a] | - | - | n.d. | - | n.d. | - | - | n.d. | - | n.d. | - | n.d. | - | n.d. | + | n.d. | - |
| 74[a] | - | - | n.d. | - | n.d. | - | + | - | - | n.d. | + | n.d. | - | n.d. | + | n.d. | + |
| 75[a] | - | - | n.d. | - | n.d. | - | - | n.d. | - | n.d. | - | n.d. | - | n.d. | + | n.d. | - |
| 76 | - | - | n.d. | - | n.d. | - | - | n.d. | - | n.d. | - | n.d. | - | n.d. | n.d. | + | - |
| 77 | - | - | n.d. | - | n.d. | - | - | n.d. | - | n.d. | - | n.d. | - | n.d. | n.d. | + | - |
| 78 | - | - | n.d. | - | n.d. | - | - | n.d. | - | n.d. | - | n.d. | - | n.d. | n.d. | + | - |
| 79 | - | - | n.d. | - | n.d. | - | + | - | - | n.d. | +/- | - | +/- | [A] | n.d. | + | - |
| 80 | - | - | n.d. | - | n.d. | - | - | n.d. | - | n.d. | - | n.d. | - | n.d. | n.d. | + | - |

ICT: immunochromatographic test; POC: point of care; ELISA: enzyme-linked immunosorbent assay; IFAT: immunofluorescence antibody test; WB: Westernblot; IHA: indirect hemagglutination test;

[A] in the case of a positive or inconclusive ELISA result, the identical ELISA was repeated and the mean was calculated; deviation of both values ≤ 20%. ;

[B] in the case of a positive or inconclusive ELISA result, a second ELISA based on a different antigen preparation was used as confirmatory test;

+: positive test result; –: negative test result; +/– : inconclusive test result; n.d.: not done;

in grey: interpreted as overall true positive test result;

* parasitological confirmed infection by detection microfilaria in blood/*Strongyloides* larvae in stool samples.
