# Supplementary figures and images for: Performance of a rapid immuno-chromatographic test (Schistosoma ICT IgG-IgM) for detecting Schistosoma-specific antibodies in sera of endemic and non-endemic populations
Source: PLoS Negl Trop Dis. 2022 May 27;16(5):e0010463. doi: 10.1371/journal.pntd.0010463 (PMC9212132; doi:10.1371/journal.pntd.0010463)

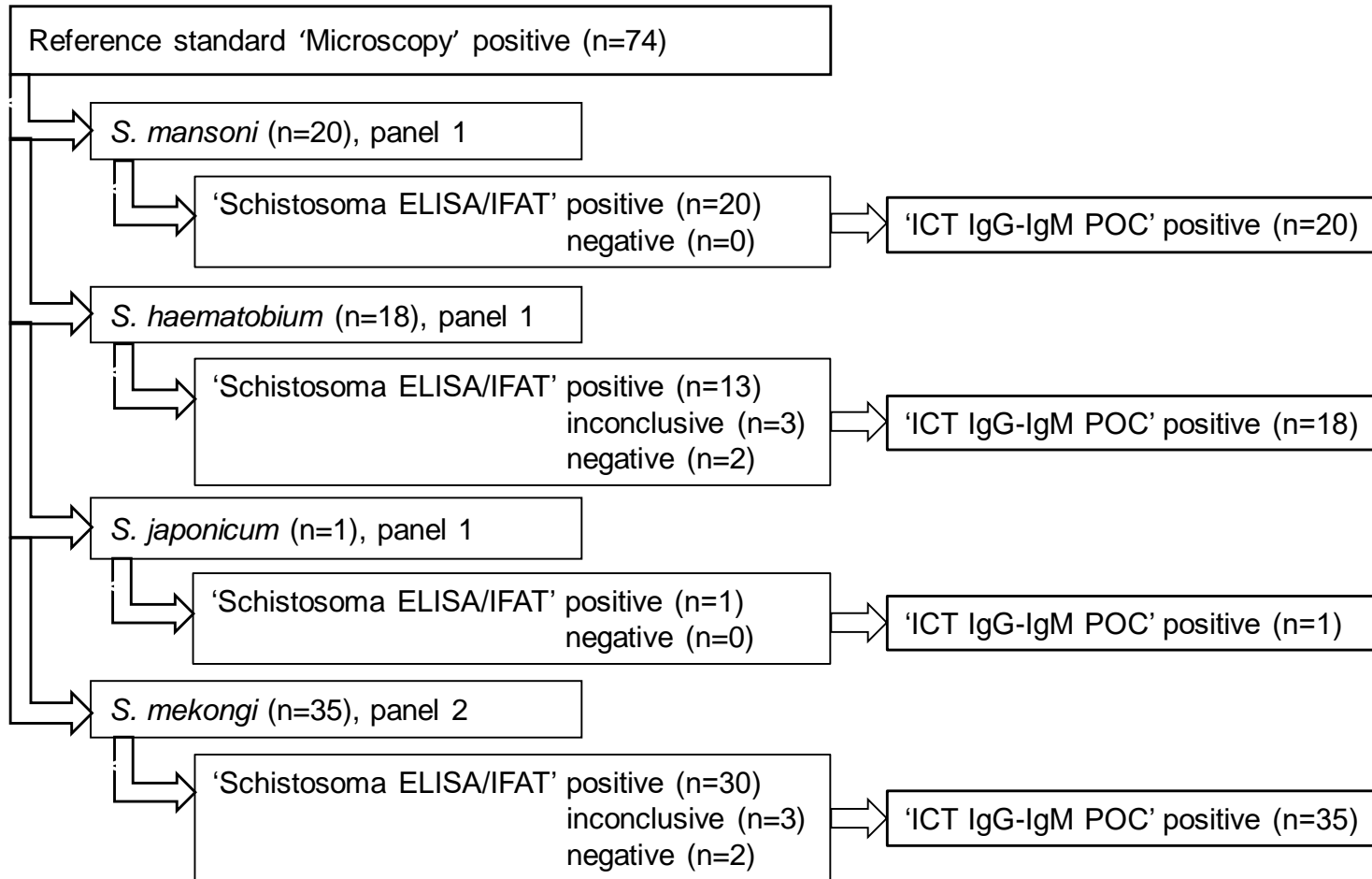

**S1 Fig.** Diagram with *Schistosoma* positive Sera of Panel 1 and 2.

Supplement: S1 Fig — (PDF) [file pntd.0010463.s003.pdf]

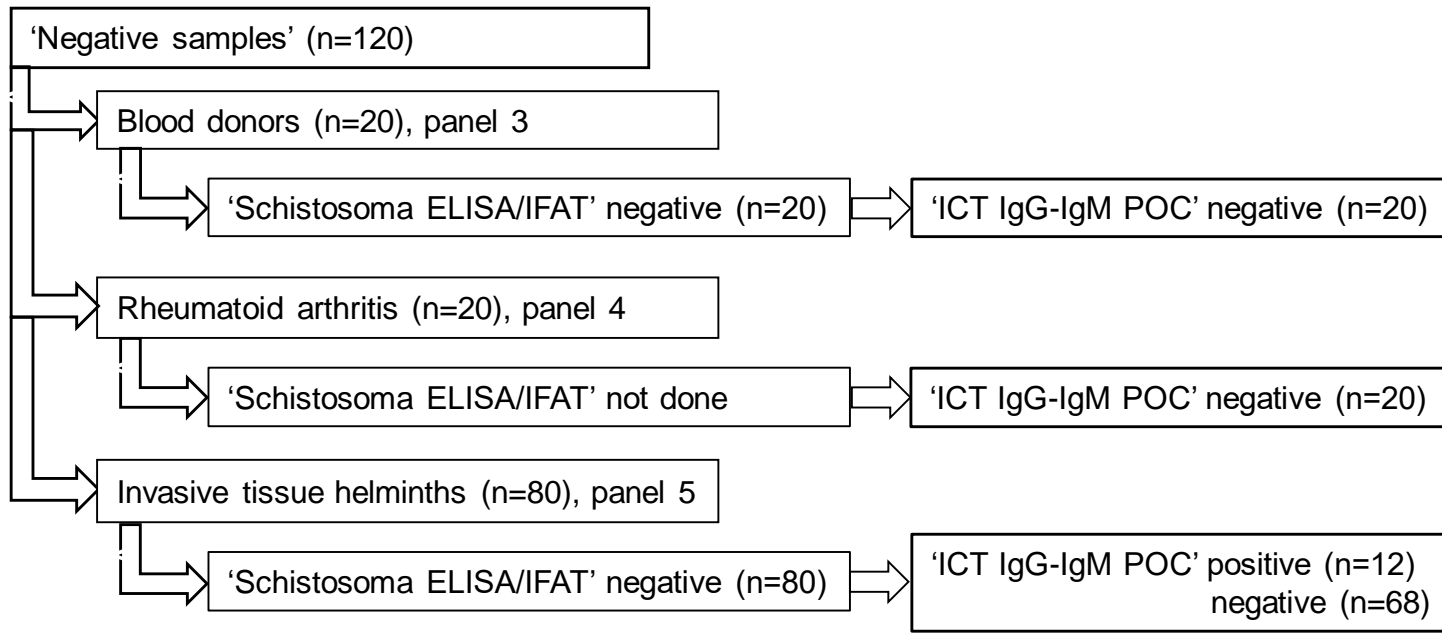

**S2 Fig.** Diagram with *Schistosoma* negative Sera of Panel 3, 4 and 5.

Supplement: S2 Fig — (PDF) [file pntd.0010463.s004.pdf]
